# Hemostatic Balance in Severe Trauma

**DOI:** 10.3389/fped.2020.600501

**Published:** 2020-11-11

**Authors:** Thorsten Haas, Melissa M. Cushing

**Affiliations:** ^1^Department of Pediatric Anesthesia, Zurich University Children's Hospital, Zurich, Switzerland; ^2^Department of Pathology and Laboratory Medicine, Weill Cornell Medicine, New York, NY, United States

**Keywords:** trauma, massive bleeding, thrombosis, viscoelastic testing, transfusion, pediatrics, coagulation factor concentrates

## Abstract

Acute coagulopathy is prevalent in adult and pediatric trauma patients and is associated with increased morbidity and mortality. While reasonable hypotheses have been created to explain the underlying perturbations of adult trauma coagulopathy (i.e., tissue factor-related increase in thrombin generation, protein C activation, hypoperfusion, and hyperfibrinolysis), only a small number of studies have been performed to prove whether these mechanisms can likewise be detected in pediatric trauma patients. In addition, severe hypofibrinogenemia (<100 mg/dL) is a frequent finding in pediatric trauma patients (>20%). Although the probability of life-threatening coagulopathy is low with minor to moderate injury, it is present in almost all patients with an injury severity score >25, hypotension, hypothermia, and acidosis. As these multifactorial changes in hemostasis cannot be adequately and rapidly measured using standard laboratory testing, the use of viscoelastic measurements has been established in adult trauma management, but prospective studies in children are urgently needed. Apart from diagnostic challenges, several studies have focused on the impact of blood product ratios on the treatment of massively bleeding pediatric trauma patients. The majority of these studies were unable to show improved survival by using higher plasma to red blood cell ratios or higher platelet to red blood cells ratios, but there are no published randomized trials to definitively answer this question. A goal-directed transfusion protocol using viscoelastic tests together with early substitution with an antifibrinolytic and fibrinogen replacement is a promising alternative to traditional ratio-based interventions. Another crucial factor in treating trauma-induced coagulopathy is the early detection of hypofibrinogenemia, a common condition in massively transfused patients. Early treatment of hypofibrinogenemia is associated with improved morbidity and mortality in adults, but needs to be further studied in future pediatric trials. Pediatric trauma patients are not only threatened by coagulopathy-related bleeding but are also at higher risk for venous thromboembolism. Pediatric trauma patients with brain injury, central venous catheters, immobilization, or surgical procedures are at highest risk for developing a deep venous thrombosis. There are no specific pediatric guidelines established to prevent venous thromboembolism in children suffering from traumatic injury.

## Introduction

Adult traumatic coagulopathy is a commonly studied, but not fully understood, condition that occurs early and is a major contributor to injury-related mortality (1, 2). Its presence is usually defined by detection of impaired plasmatic coagulation parameters, such as a prolonged prothrombin time (PT) or activated partial thromboplastin time (aPTT) or increased International Normalized Ratio (INR). The occurrence of a prolonged PT or increased INR (specifically INR of ≥1.3 at admission) is associated with mortality in trauma patients (3, 4). Although INR seems to predict mortality, prospective data from adult surgical and trauma patients have shown that INR is not a reliable indicator of coagulopathy and should not be used to guide factor replacement (5).

While initial theories to describe trauma-related coagulopathy were based on the effects of hemodilution due to the administration of large amounts of coagulation factor-free solutions, the knowledge about the pathophysiologic changes have evolved substantially. The current understanding of trauma-induced coagulopathy is still evolving, but the main underlying causes are thought to be hemorrhagic shock-related hypoxia/hypoperfusion, and tissue injury. Hypoperfusion and hypoxia promote platelet and endothelial dysfunction that further trigger activation of protein C and impaired thrombin generation, as well as major changes in the fibrinolytic pathway (6). In addition, defects in collagen deposition related to endothelial dysfunction, downregulated platelet glycoprotein receptors, and complement activation are all thought to be contributors to Trauma Induced Coagulopathy (TIC) (7). In a prospective trial in pediatric trauma patients with an age of >15 years, the probability of life-threatening coagulopathy was low with minor to moderate injury, but coagulopathy was present in almost all patients with an injury severity score >25, hypotension, hypothermia, and acidosis (8). It is unknown whether the coagulopathy is similar for children in all age groups suffering from trauma due to a dearth of prospective studies in pediatric populations.

In addition to a brief description of TIC in children and discussion of the major differences between hemostatic changes in adults and children suffering from trauma, this review will focus on the management of coagulopathic bleeding in pediatric trauma patients. Finally, we will review the impact of trauma on the occurrence of venous thromboembolism, as this is an important but frequently overlooked counterpart of trauma-related bleeding complications.

## Trauma-Induced Coagulopathy in Children

### Overview of TIC in Children

The incidence of TIC in severe pediatric trauma patients in the emergency room varies tremendously and is reported to be in the range of 10–77% (9). This remarkable variability may be explained by the fact that to date no specific pattern of tests and no specific thresholds are available to define TIC. There is no universally accepted definition of TIC. In fact, Christiaans et al. (9) described a huge variety of coagulation parameters and thresholds from 18 different studies that were used to define TIC after pediatric trauma (9), thus questioning if the main results of those studies are comparable. In addition, it is known from the adult world that changes in hemostasis following trauma do not uniformly lead to bleeding. There may be transient changes that lead to hypercoagulability as well.

Although the TIC-related changes in hemostasis and fibrinolysis of severely injured adult patients can be likewise observed in children (10), age-dependent differences may occur due to developmental changes. The overall hemostatic balance in an infant prevents an increase in bleeding or hypercoagulable states; however, it is hypothesized that even small changes in this fragile balance may lead to a coagulopathy in one direction or the other depending on the type of injury or time period after injury (10).

### The Role of Hypercoagulability and Fibrinolytic Pathway Abnormalities in Pediatric Trauma-Induced Coagulopathy

In adult trauma patients, the presence of hyperfibrinolysis is highly correlated with mortality (11). Fibrinolysis is also a concern in children. In a cohort of 133 pediatric trauma patients, 19.6% of all children presented with hyperfibrinolysis detected by rapid thrombelastography. In addition, 38.3% of the children had fibrinolytic shutdown (12). In that study, hyperfibrinolysis and fibrinolytic shutdown were both associated with mortality and the need for blood transfusion, while fibrinolytic shutdown was additionally associated with disability (e.g., changes in behavior, intellectual functioning, and locomotion) and deep vein thrombosis. A prospective follow up study performed by the same group demonstrated that plasma transfusion and TBI are both independently associated with fibrinolysis shutdown, and that the combination of both is associated with a very poor prognosis, showing a 75% mortality and 100% disability in all survivors (13). Based on those results, the authors concluded that TIC is not a single entity, but a broad group of hemostatic imbalances showing distinct phenotypes.

While the empiric administration of tranexamic acid to address hyperfibrinolysis has shown to reduce mortality in adult trauma patients, data in pediatric trauma are scarce (14). Studies are urgently needed to investigate whether preadmission administration of tranexamic acid in pediatric civilian trauma patients has an impact on bleeding and mortality. There are no studies that address the treatment of fibrinolytic shutdown in children.

### Pathophysiology of the Mechanisms of Pediatric Trauma (Including Traumatic Brain Injury)

There is a substantial difference in the underlying mechanisms of trauma in children as compared to adults: a relatively higher rate of blunt trauma and non-accidental trauma, including brain injuries, can be observed in children (15). This is of particular importance as isolated severe brain injury (TBI) is a major contributor to the development of trauma-induced coagulopathy (TIC), and TIC can be observed in about 42–44% of all severe TBI cases (16, 17). Indeed, pediatric TBI has been identified as a common contributor to TIC and mortality (18). In adult patients suffering from TBI, an early transition from a hyper- to a hypocoagulable state has been observed (19, 20). Zhang et al. (21) have demonstrated in a mouse model that the release of brain-derived cellular microvesicles into the circulation following brain trauma has served as a causal factor for induction of a systemic hypercoagulable state, which was quickly followed by a consumptive coagulopathy. Focusing on the time course of traumatic brain injury-related coagulopathy, D-dimers and fibrinogen degradation products can be detected within minutes of injury, whereas prolonged PT and aPTT are detected late, reaching their peaks ~3–6 h post-TBI (19, 20, 22). The combination of both, a hyper- and hypocoagulable state have been likewise detected in pediatric TBI (23). Hypercoagulability is suspected to promote ischemic lesions which can further worsen the outcome in this setting (9).

### Summary

In summary, when TIC is present in pediatric patients the mortality rate appears to be markedly increased (3, 24). Unfortunately, the definition of TIC is not yet fully understood, especially in children, and may encompass several distinct abnormal hemostatic profiles. Therefore, a timely and reliable diagnostic workup is a basis for a better understanding of the hemostatic balance and optimal bleeding management. This will be discussed in the next section.

## Detection of Trauma-Induced Coagulopathy in Children

In addition to reviewing global plasmatic coagulation parameters (i.e., PT/INR and aPTT) which provide only a simple assessment of the first phase of clot initiation, it seems worthwhile to review published evidence of other markers to detect TIC, such as platelet count, fibrinogen, signs of fibrinolysis and viscoelastic testing.

Platelet count was significantly associated with mortality in a cohort of 102 pediatric trauma patients, and an abnormal platelet count was observed in 23% of all children (15). However, platelet count does not reflect platelet function, which may also play a role in TIC (25). In adult patients with TBI, platelet ADP receptor inhibition was strongly correlated with the severity of brain injury (6). Unfortunately, sound data in children are lacking to determine the impact of platelet count and function on TIC, and clear thresholds are needed to safely guide transfusion therapy.

Severe hypofibrinogenemia (plasma fibrinogen levels below 100 mg/dL) was detected in 20% of the previously mentioned cohort of pediatric trauma patients (15). It remains unclear if this was due to hemodilution or consumptive coagulopathy on large wound surfaces, or because of hyperfibrinolysis.

When viscoelastic testing such as thrombelastography or thromboelastometry was used to detect TIC in pediatric trauma patients, signs of hyperfibrinolysis (defined as lysis index of 3.0% or greater or maximum lysis >15%, respectively) (12), a prolonged time for initiation of clot building (R-time and k-time, or clotting time and clot formation time, respectively), as well as signs of weakening in clot strength (maximum amplitude or maximum clot firmness, respectively) were observed (26, 27). Signs of TIC using standard coagulation testing and viscoelastic testing are shown in Table 1.

**Table 1:** Signs of trauma-induced coagulopathy using standard laboratory testing (SLT) and viscoelastic testing (VET).

**Characteristics**	**Signs in SLT**	**Signs in VET**
Impaired initiation of coagulation	Prolonged aPTT Prolonged PT/elevated INR	Prolonged R-time (TEG) Prolonged CT (ROTEM)
Weakening of clot strength	*Not measurable*	Reduced MA (TEG) Reduced MCF (ROTEM)
Hypofibrinogenemia	Reduced value in Clauss fibrinogen assay	Reduced MA FF (TEG) Reduced FIBTEM MCF (ROTEM)
Hyperfibrinolysis	*Not measurable*	LY30 ≥ 3% (TEG) ML > 15% (ROTEM)

*aPTT, activated partial thromboplastin time; PT, prothrombin time; INR, international normalized ratio; TEG, thrombelastography; ROTEM, thromboelastometry; R-time, reaction time; CT, clotting time; MA, maximum amplitude; MCF, maximum clot firmness; FF, functional fibrinogen test; FIBTEM, fibrin polymerization assay; LY30, Clot lysis at 30 min after maximum clot strength; ML, maximum lysis at 60 min after CT*.

## Pediatric Transfusion Strategies

There is published evidence that hemorrhagic injuries are less likely in children, and TBI is the main underlying reason for mortality. In a cohort of 776 pediatric trauma patients, clinical bleeding or hemorrhagic injury was only present in 7.0% (*n* = 54) of all children (4). Although in that study an admission INR of ≥1.3 was determined to be an independent predictor of mortality, transfusion of plasma was not effective in changing INR. The authors have therefore questioned whether an INR of 1.3 or greater can be used as a precise term to diagnose coagulopathy in this setting or whether it is just an indicator of disease severity. It is of great importance to understand and to distinguish the interpretation of impaired plasmatic coagulation test results as markers of systemic hemostasis imbalance and outcome predictors vs. our ongoing efforts to find reliable laboratory treatment targets for transfusion therapy. Notably, patients with borderline elevated plasmatic coagulation tests do not necessarily show signs of disturbed clot initiation in viscoelastic tests (28, 29). As mentioned before, transfusion of plasma to treat a moderately elevated INR is not successful and does not have influence on outcome (4). Therefore, the transfusion strategy should be adopted primarily based on the clinical status of a trauma patient, namely by whether clinically relevant bleeding is present or not. A threshold of 37 ml/kg per 4 h or more of transfused blood was determined to be an indicator for the need for hemorrhage control procedures and a predictor of early mortality (30).

If clinically meaningful hemorrhage is present, immediate access to blood and blood products is needed. Institutional protocols should be established. However, there is no evidence to support transfusion protocols using a 1:1:1 approach of red blood cells (RBCs), plasma and platelets in pediatrics. Unlike in the adult setting, several trials have failed to show any survival benefit by using a transfusion protocol with a predefined transfusion ratio (31–37). In a retrospective analysis of a cohort of 364 massively transfused combat-injured children, a high ratio of plasma to RBC transfusion was associated with a longer hospital length of stay and was not associated with a mortality benefit (36). When comparing a cohort of 55 pediatric trauma patients that were transfused with a protocol using a predefined massive transfusion ratio, Chidester et al. (31) were not able to show a significant difference in mortality. There is only one retrospective data analysis that found a benefit. In this study of 465 pediatric trauma patients that excluded severe isolated head injury, a survival benefit was detected for children treated with a high ratio of plasma:RBC, while no differences were observed among the higher platelet:RBC cohort (38). Given that there are major differences in terms of definition of coagulopathy and criteria to activate a massive transfusion protocol in different published studies, it is hardly surprising that there is no clear and easy answer as to whether certain transfusion ratios may have a mortality benefit in civilian pediatric trauma patients. In addition to study differences, differences in age groups, the frequency of severe brain injury, as well as inconsistent adherence to transfusion protocols and laboratory thresholds among published trials also cloud the picture.

Recently, the successful resuscitation using whole blood at a dose of 20 ml/kg was reported in a cohort of 18 pediatric civilian trauma patients (39). However, more evidence is urgently needed before the use of whole blood can be recommended in children.

## Goal-Directed Hemostatic Resuscitation

**Figure 1:**
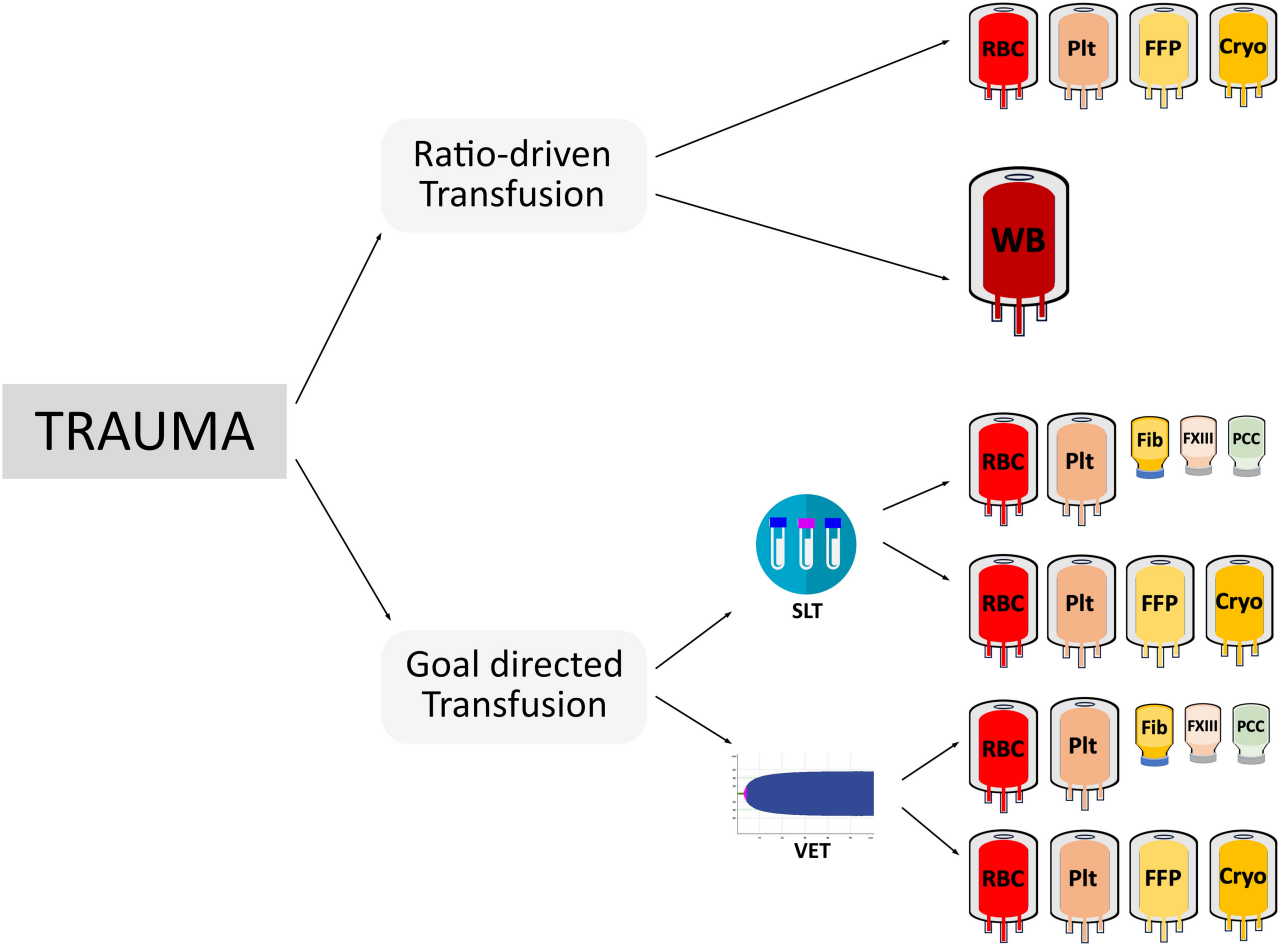
This figure illustrates the various potential treatment options for acutely bleeding trauma patients depicting the considerable differences in coagulation testing (SLT, standard laboratory testing; VET, viscoelastic testing), treatment options (ratio-driven vs. goal-directed transfusion), blood components (RBC, red blood cells; FFP, plasma; PLT, platelet concentrates; or CRYO, cryoprecipitate), the use of whole blood (WB), or coagulation factors (Fib, fibrinogen concentrate; PCC, prothrombin complex concentrate; FXIII, factor XIII concentrate).

As there is no clear evidence to support ratio driven transfusion protocols in pediatric trauma patients, goal-directed transfusion management using viscoelastic testing offers an alternative approach. Data from an adult pragmatic randomized trial in 111 trauma patients demonstrated that a group of patients whose treatment was guided by thrombelastography had improved survival compared to patients whose treatment was guided by conventional coagulation tests (40). Higher blood product utilization, with more units of plasma and platelets in the first 2 h of resuscitation, occurred in the group guided by conventional coagulation tests. Survival in the thrombelastography group was significantly higher than the conventional coagulation test group. In another adult trauma trial that compared thromboelastometry-guided transfusion management with purified concentrates compared to plasma transfusion vs. a targeted administration, the former group showed superior outcome (41). Not only was the amount of transfused allogeneic blood products smaller in the coagulation factor group, but the time to correct TIC and improve bleeding was significantly shorter in the coagulation factor-group (median of 22.5 min [IQR; 13.5–40.0] vs. 128.0 min [IQR; 48.3–186.3]) vs. the plasma group (estimated time difference of −97 min [−126 to −60], *p* < 0.0001). Another important finding of that study was that almost all patients suffered from severe hypofibrinogenemia after arrival at the emergency room, which was successfully treated by administration of fibrinogen concentrate at a dose of 50 mg/kg, while a dose of 30 ml/kg of plasma failed to normalize plasma fibrinogen levels. The importance of detecting and treating hypofibrinogenemia early was also investigated in a prospective cohort study of 517 adult trauma patients (42). Multiple studies have demonstrated evidence that hypofibrinogenemia is a critical issue in severe bleeding for pediatric intraoperative and trauma patients (43–45). One study in pediatric trauma patients analyzed an institutional transfusion protocol where cryoprecipitate was not included until the third transfusion package. The authors found that 11% of all cases had fibrinogen levels below the detection limit of the assay upon arrival in the emergency department. They concluded that future renditions of their Massive Transfusion Protocol (MTP) would have included cryoprecipitate earlier. Plasma is not adequate to replenish low fibrinogen levels (32). Viscoelastic testing guided bleeding management could guide earlier fibrinogen replacement since acquired hypofibrinogenemia can be detected within 5–10 min. However, the search for a perfect bleeding management approach is hampered by a marked difference in methodologies between published trials. Trials have used different coagulation tests (conventional or viscoelastic testing) and applied different treatment options (ratio-driven transfusion, administration of coagulation factors, or a mix of both), thus leading to a variety of alternative approaches (Figure 1).

**Figure 2:**
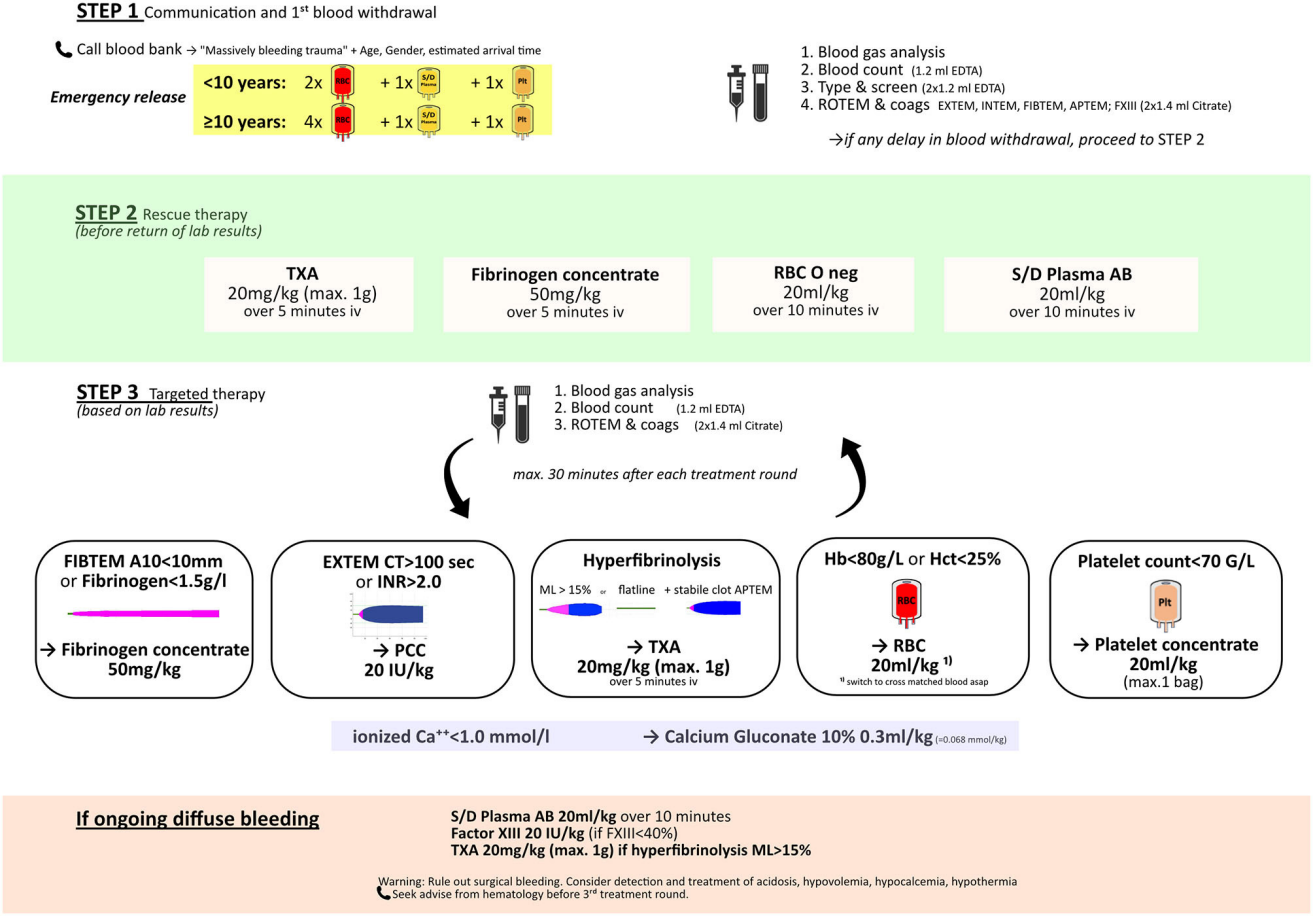
Example of a goal-directed pediatric bleeding management algorithm. TXA, tranexamic acid; RBC, red blood cells; S/D plasma AB, solvent/detergent pooled plasma, blood group AB.

An example of a goal-directed pediatric bleeding management algorithm is displayed in Figure 2. This approach is feasible even in young children. The combination of viscoelastic testing plus resuscitation with factor concentrates is appealing as results are rapidly available to the trauma team and the preparation of coagulation factors can be performed extremely quickly. Furthermore, the lower volume and pathogen-inactivated status of coagulation factors compared to standard blood components are important for safety in pediatric patients. Rapidly available testing and blood components are of particular importance as the management of pediatric trauma patients is further challenged by difficulties and delays in establishing (large bore) venous access for phlebotomy and fluid resuscitation.

## Venous Thromboembolism in Trauma

Children sustaining severe traumatic injuries are not only threatened by the risk of bleeding, but also have an increased risk for developing venous thromboembolism (VTE). Although very young children tend to be protected from VTE by their quantitative and qualitative developmental differences in hemostasis (46), the risk factors for VTE in older pediatric patients are well-established. In addition to individual inherited or acquired risk factors for thrombosis, multiple disease- or treatment-related risk factors contribute to VTE (47). The incidence of VTE in pediatric trauma patients is <0.5% (48–50). This is in sharp contrast to the adult trauma patient population where the estimated incidence is as high as 20–58% without appropriate thromboprophylaxis (47). However, there is a paucity of evidence for standardized VTE screening in children and thus children are monitored for VTEs less frequently so the actual incidence may differ. Additional risk factors such as major surgery, placement of a central venous line, injury severity score, age, poor perfusion with necessity for inotrope support, Glasgow coma scale, intubation, and blood transfusion have been identified as contributors to VTE risk.

Although there is currently no consensus for routine administration of thromboembolism prophylaxis in the pediatric trauma setting, a group from Johns Hopkins created their own VTE prediction score based on risk factors identified in their risk assessment model (50). A retrospective review of a pediatric trauma registry encompassing 13,880 patients found no VTE in the age group < 13 years, two VTEs in the age group 13–17 years, and 59 VTEs in the age group > 17 years old (51). A consensus conference regarding the prevention of VTE in pediatric trauma patients judged current scientific data as insufficient for evidence-based decisions. However, they provided the recommendation that injured children < 12 years old do not routinely need VTE prophylaxis, but prophylaxis should be considered in children with a history of VTE, and may be considered in children with a central venous catheter (52). A recently published retrospective cohort of 209 pediatric trauma patients showed that femoral venous catheter-associated thrombosis is significantly more frequent than in non-femoral catheters (53). When thromboembolism prophylaxis with low-molecular-weight heparin was administered to a cohort of 706 pediatric trauma patients, major bleeding was reported in 0.4% of those children, while thromboembolic events were detected in 2.1% (54).

## Conclusions and Future Directions

At present, the knowledge regarding hemostatic balance in severe pediatric trauma patients is mostly extrapolated from adults. More studies are urgently needed to understand the pathophysiologic changes in this patient population. Trauma-induced coagulopathy is not just one single entity, but a heterogenous group of hemostatic imbalances showing distinct phenotypes depending on timing, mechanism of injury, underlying conditions and other less understood factors. The conditions are even more heterogeneous in children, where the age of the children impacts the mechanism of injury as well the baseline hemostatic factors. As such, a rapid, frequent and reliable laboratory testing should be performed to detect the current and evolving clinical picture. Coagulation tests such as INR or PT may not accurately assess the hemostatic imbalance and may lead to over transfusion of plasma without improving the outcome. The implementation of a viscoelastic testing protocol has been beneficial in adult trauma treatment and should be validated in the pediatric setting. A targeted transfusion approach may offer a feasible alternative in pediatric bleeding management, with focus on the treatment of hyperfibrinolysis and hypofibrinogenemia. Although thromboembolism prophylaxis is not generally recommended in pediatric trauma patients < 12 years old, future studies should investigate whether viscoelastic testing can be used to predict thrombosis, thus giving a more global picture of hemostatic balance in this patient population.

## Author Contributions

TH and MC conceptualized and wrote the manuscript. Both authors contributed to the article and approved the submitted version.

## Conflict of Interest

TH has received lecturer's fees and travel support from Octapharma and Instrumentation Laboratory, and is a consultant for Octapharma. MC is a consultant for Cerus Corporation and Octapharma.
